# Age-dependent associations between telomere length and environmental conditions in roe deer

**DOI:** 10.1098/rsbl.2017.0434

**Published:** 2017-09-27

**Authors:** Rachael V. Wilbourn, Hannah Froy, Marie-Christina McManus, Louise Cheynel, Jean-Michel Gaillard, Emmanuelle Gilot-Fromont, Corinne Regis, Benjamin Rey, Maryline Pellerin, Jean-François Lemaître, Daniel H. Nussey

**Affiliations:** 1 Institute of Evolutionary Biology, University of Edinburgh, Edinburgh EH9 3FL, UK; 2 CNRS, Université Lyon 1, Laboratoire de Biométrie et Biologie Evolutive UMR5558, 69622 Villeurbanne, France; 3 Office National de la Chasse et de la Faune Sauvage, Unité Cervidés-Sanglier, Bar-le-Duc, France

**Keywords:** leucocyte, wild mammal, body mass, condition, biomarker

## Abstract

Telomere length (TL) represents a promising biomarker of overall physiological state and of past environmental experiences, which could help us understand the drivers of life-history variation in natural populations. A growing number of studies in birds suggest that environmental stress or poor environmental conditions are associated with shortened TL, but studies of such relationships in wild mammals are lacking. Here, we compare leucocyte TL from cross-sectional samples collected from two French populations of roe deer which experience different environmental conditions. We found that, as predicted, TL was shorter in the population experiencing poor environmental conditions but that this difference was only significant in older individuals and was independent of sex and body mass. Unexpectedly, the difference was underpinned by a significant increase in TL with age in the population experiencing good environmental conditions, while there was no detectable relationship with age in poor conditions. These results demonstrate both the environmental sensitivity and complexity of telomere dynamics in natural mammal populations, and highlight the importance of longitudinal data to disentangle the within- and among-individual processes that generate them.

## 1. Introduction

Understanding how environmental variation shapes organismal physiology and life history in wild systems is fundamental to evolutionary ecology, but identifying physiological biomarkers relevant to life history and fitness is challenging. Recently, telomere length (TL) has emerged as a potential biomarker of an individual's physiological state and past environmental experiences [1]. Telomeres are repetitive DNA segments that maintain genomic integrity by capping the ends of eukaryotic chromosomes and forming complexes with proteins [2]. Telomeres shorten with each cell division and are sensitive to oxidative damage, and critically short telomeres trigger cellular senescence *in vitro* [3]. In humans, average TL decreases with age and short TL in adulthood predicts late-onset disease and mortality [3], while past experience of stressful events is associated with shortened adult TL [4]. In birds, short TL predicts increased mortality risk [5], and experimentally induced competition for food or physiological stress accelerates telomere attrition during early life [6,7]. Accordingly, there is growing interest in how natural variation in environmental conditions influences telomere dynamics, and recent studies in wild birds and fishes suggest that populations experiencing physiologically challenging environments, particularly in early life, have shorter TL [8–12]. Although associations between TL, age, sex and survival have recently been reported in wild mammals [13–15], the relationship between environmental conditions and mammalian telomere dynamics is currently unknown.

Here, we test how broad differences in environmental conditions influence telomere dynamics in a wild mammal by comparing patterns of TL variation between two populations of European roe deer (*Capreolus capreolus*). These populations experience markedly different environmental conditions [16], with consequences for various life-history and demographic parameters [17], as well as body condition and immune phenotype [18]. Based on trends emerging from the recent literature on humans and birds, we hypothesized that persistent experience of a poor environment would result in shorter TL at any given age, owing to lower initial TL in early life and a faster rate of TL shortening over an animal's lifetime. Since body mass at a given age is thought to reflect overall physiological condition in this species [18], we also predicted a positive association between body mass and TL within populations.

## 2. Methods

Blood samples were collected from roe deer at two long-term study sites that differ markedly in environmental conditions (January–March 2016). Both deciduous woodland habitats, the Trois-Fontaines site (TF; 1360 ha) in northeastern France (48°43′ N, 4°55′ E) has more fertile soils, a continental climate and higher forest productivity than Chizé (CH; 2614 ha) in western France (46°50′ N, 0°25′ W). Poor-quality soils and summer droughts at Chizé result in low forest productivity [16], and deer living there consequently have reduced growth rates, adult size and fecundity, and markers of physiological condition [17,18]. Body mass was taken at capture, and all individuals were of known age and sex (TF: 34 females, 39 males; CH: 36 females, 30 males). Buffy coat fractions, comprising mainly leucocytes, were prepared in the field and immediately frozen at −80°C until DNA extraction. Relative TL was measured by quantitative PCR as described previously [19] and in the electronic supplementary material.

We ran linear models in R v.3.3.3. We tested our first hypothesis by running a model of TL including sex and population as two-level factors, age as a linear covariate and all possible two-way interactions among these terms. A backward elimination approach was used to remove non-significant terms from the maximal model. We used a similar approach to confirm previously established differences in body mass between the two populations, while accounting for the effects of sex and age (linear and quadratic terms). Finally, we tested whether a measure of body condition explained variation in TL, independent of its associations with age, sex and population, by adding size-corrected body mass (residuals from a regression of body mass on hind foot length, see electronic supplementary materials) as a covariate to the minimal model for TL, and applying the same backward simplification.

## 3. Results

**Figure 1.**
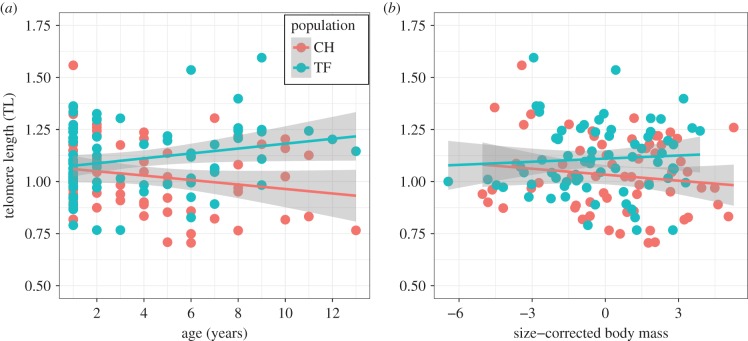
Changes in relative leucocyte telomere length (TL) with age (*a*) and size-corrected body mass (*b*) in two different populations of roe deer. Raw data for CH (red) and TF (blue) are presented with a linear regression (red and blue lines, respectively) and associated standard errors (grey shading). (Online version in colour.)

There was an interaction between the effects of age and population on TL (*F*_1,135_ = 6.294, *p* = 0.013; electronic supplementary material, table S1): shorter telomere lengths were observed in the poor environment of CH but only among older individuals (figure 1*a*). This was underpinned by a marginally non-significant increase in TL with age in TF (*F*_1,71_ = 3.849, *b* = 0.012 ± 0.006 s.e., *p* = 0.054) and non-significant decline with age in CH (*F*_1,64_ = 2.562, *b* = −0.011 ± 0.007 s.e., *p* = 0.114). There was no evidence for sex differences in TL, or interactions between sex and age or population (electronic supplementary material, table S1). Individuals from TF were heavier regardless of age or sex (electronic supplementary material, figure S1 and table S2), as has been previously documented [17]. There was no evidence for a relationship between size-corrected body mass and TL (figure 1*b*; electronic supplementary material, table S3), and including size-corrected mass in the TL model did not alter the magnitude of the age-by-population interaction (electronic supplementary material, table S3).

## 4. Discussion

In this study, we provide, to our knowledge, the first evidence for contrasting telomere dynamics in wild mammal populations experiencing different environmental conditions. As predicted, we found shorter TL in the population experiencing a poorer environment, but this difference was only apparent at older ages and was underpinned by a cross-sectional increase in TL with age in the population experiencing good environmental conditions. Our study adds to an emerging literature on wild birds and fishes documenting associations between TL and environmental conditions, although most studies have focused on early life. For instance, experimental brood enlargements, expected to increase competition for parental provisioning and physiological stress in developing birds, are associated with shortened TL [7], and being raised in urban or higher altitude populations reduced TL in nestlings [8,10]. Likewise, in salmon, young-of-the-year from higher average temperature rivers (i.e. higher thermal stress) had shorter TL [9]. By contrast, we found no evidence of a difference in TL among roe deer populations in the youngest age groups, despite fawns in different locations experiencing marked differences in climatic conditions and food availability *in utero* and during early life [17]. Previous studies in birds have detected shorter TL in adults experiencing more challenging environments [11,12]. However, our data encompass the full natural age range in both populations and imply that the environmental effect on TL is a cumulative one that is apparent only later in adulthood at the population level.

We predicted, assuming that increased environmental stress drives more rapid telomere attrition, that declines in TL with age should be greater in CH than TF. However, TL actually increased with age in TF and tended to decline in CH. There is growing appreciation that within-individual lengthening of TL can and does occur [20], although the process remains poorly understood. Cross-sectional changes in telomere length, however, are not necessarily driven by within-individual changes, and the selective disappearance of individuals with short telomeres has been observed to increase average TL with age in wild mammals [13]. It is possible that both our study populations are experiencing selective disappearance, but that poor environment at CH may drive more rapid TL shortening compared with TF, making the increase in TL in older individuals not detectable in this population. Overall, our results highlight the potential complexity of telomere dynamics in natural systems, and the importance of long-term longitudinal studies to disentangle the contributions of within- and among-individual processes to these dynamics.

We found no evidence that size-corrected body mass was associated with TL in either study population (figure 1*b*), despite marked differences in average body mass across all ages between populations (electronic supplementary material, figure S1). We predicted a positive relationship between TL and body mass within populations, but note that previous studies in birds and mammals have reported conflicting associations between TL and either early-life growth rates or body mass [14,15]. A previous study comparing the same two roe deer populations found that while CH had lower levels of metabolic markers (e.g. haemoglobin and albumin levels) associated with body condition than TF, immunological markers were not consistently lower at CH [18]. In contrast to other vertebrates, mammals have enucleated red blood cells and TL measurements from blood only include leucocytes, which means immune status could have a much greater influence on telomere dynamics. Although a recent study of Soay sheep found little evidence that leucocyte TL and leucocyte cell composition were associated [14], the role of infection history and immune phenotype in the population differences in TL reported here remains to be determined.

We have presented important evidence for sex and body mass independent differences in TL among populations experiencing contrasting environments. The crucial next step for the application and understanding of TL as a biomarker in wildlife ecology will be to understand the particular aspects of environmental conditions and physiological status that TL responds to and how these in turn relate to life history and fitness.

## Supplementary Material

Supplementary material

## Supplementary Material

Data

## Data accessibility

Data available as electronic supplementary material.
