# Supplementary material for: Age-dependent associations between telomere length and environmental conditions in roe deer
Source: Biol Lett. 2017 Sep 27;13(9):20170434. doi: 10.1098/rsbl.2017.0434 (PMC5627176; doi:10.1098/rsbl.2017.0434)

**Supplementary materials for:**

Age-dependent associations between telomere length and environmental conditions in roe deer

Wilbourn R.V., Froy, H., McManus, M.C, Cheynel, L., Gaillard, J.M., Gilot-Fromont, E., Regis, C., Rey, B., Pellerin, M., Lemaître, J.F. & Nussey, D.H.

**SUPPLEMETARY METHODS**

**Study population and sample preparation**

Roe deer have been continuously monitored at the Trois-Fontaines (TF) and Chizé (CH) study sites using Capture-Mark-Recapture methods since 1975 and 1977, respectively. Trapping sessions occur at each site between January and March each winter (see [1] for further details), and each year, approximately 50% of the population are captured. Sex, body mass (to the nearest 50g) and hind foot length (to the nearest mm, measured from the heel to the tip of the hoof) are recorded, and age is determined for each individual through prior knowledge of their year of birth[2]. During the 2015/2016 field season, blood samples were collected from the jugular vein of known-age individuals (n=160). Within 30 min of sampling, whole blood was spun at approximately 3000 rpm for 10 min and the plasma layer drawn off and replaced by the same quantity of 0.9% w/v NaCl solution and spun again. The intermediate buffy coat layer, comprising mainly leukocytes (white blood cells) was collected into a 1.5‐mL Eppendorf tube and stored at −80 °C until further use.

**DNA extraction**

Genomic DNA was extracted from white blood cells using Macherey-Nagel NucleoSpin® Blood QuickPure kit (Catalogue number 740569) at the CNRS, Lyon (France). All downstream processing of samples (e.g. quality control (QC), telomere measurement) was then carried out at the University of Edinburgh, Scotland (UK). DNA yield and purity was quantified using a Nanodrop ND-1000 spectrophotometer (Thermo Scientific, Wilmington DE, USA) and DNA integrity was assessed by running 200 ng total DNA on a 0.5% agarose gel and DNA bands scored on a scale of 1-5 by visual examination. Samples passed QC with a DNA yield of ≥20 ng/µl, an acceptable purity absorption range of 1.7 - 2.0 for the 260/280 nm ratio and > 1.8 for the 260/230 nm ratio, and a DNA integrity score of either 1 or 2 [3].

*DNA extraction protocol*

1. Lyse blood samples

Pipette 25 µL Proteinase K and up to 150 µL blood (leucocytes) + 50 µL PBS into 1.5ML microcentrifuge tubes. Incubate at room temperature for 1 min. Add 200 µL Lysis Buffer BQ1 to the samples and vortex the mixture vigorously (10-20s). Incubate samples at 70°C for 10 minutes.

1. Adjust DNA binding conditions

Vortex again the mixture and centrifuge few seconds at 11,000 x g. Add 200µL ethanol (96-100%) to each samples, vortex again, centrifuge again few seconds at 11, 000 x g.

1. Bind DNA

Add the samples to the NucleoSpin Blood QuickPure Columns placed in a collection tubes and centrifuge 1 min at 11,000 x g. If the samples are not drawn through the matrix completely, repeat the centrifugation at higher g-force (up to 15,000 x g). Discard collection tube with flow-through.

1. Wash & dry silica membrane

Place the Nucleospin blood quickpure column into a new collection tube (2mL) and add 350 µL Buffer BQ2. Centrifuge 3 min at 11,000 x g. If the samples are not drawn through the matrix completely, repeat the centrifugation at higher g-force (up to 15,000 x g). Discard collection tube with flow-through.

1. Elute highly pure DNA

Place the NucleoSpin blood quickpure column in a 1.5 mL microcentrifuge tube and add 50 µL prewarmed Buffer BE (70°C). Dispense buffer directly onto the silicq membrane. Incubate at room temperature for 1 min. Centrifuge 1 min at 11,000 x g.

**Telomere length measurement**

We measured relative leukocyte telomere length (RTL) using a real-time quantitative PCR method (qPCR; [4]) which has previously been optimised and validated in sheep and cattle [3]. This method measures the total amount of telomeric sequence present in a DNA sample, relative to the amount of a non-variable copy number reference gene (beta-2-microglobulin (B2M)). For telomere reactions we used the following HPLC purified primers, Tel 1b (5’-CGG TTT GTT TGG GTT TGG GTT TGG GTT TGG GTT TGG GTT-3’) and Tel 2b (5’-GGC TTG CCT TAC CCT TAC CCT TAC CCT TAC CCT TAC CCT-3’) (from Epel et al., 2004). For B2M reactions, primers were supplied by Primer Design (Catalogue number: HK-SY-Sh-900, Southampton, UK).

Using an automated liquid handling robot (Freedom Evo-2 150; Tecan) we were able to load both the DNA samples and qPCR master mix in 384 well plates; allowing us to run both telomeric and B2M reactions in separate wells but on a single plate. A separate master mix for each primer set was prepared containing 5 µl LightCycler 480SYBR Green I Master Mix (Cat # 04887352001, Roche, West Sussex, UK), 0.5 µl B2M (300 nm) primer or 0.6 µl each tel primer (900 nm), and 2 ng of sample DNA. DNA was amplified in 10 µl reactions. Each plate included a non-treated control (water; NTC), a calibrator sample (2ng) on each row to account for plate to plate variation and robot pipetting error, as well as a 1:4 serial dilution starting at 10ng/µl to visually inspect the qPCR curves. The calibrator sample is DNA that has been extracted from a large quantity of blood obtained from a single wild roe deer. In this case, the calibrator was extracted using the Qiagen DNeasy Blood and Tissue kit (Cat# 69581, Manchester, UK), pooled and quality controlled in the same way as our DNA samples of interest. All samples, calibrators and NTC’s were run in triplicate and all qPCR performed using a Roche LC480 instrument using the following reaction protocol: 10 min at 95 ^o^C (enzyme activation), followed by 50 cycles of 15 s at 95 ^o^C (denaturation) and 30 s at 58 ^o^C (primer annealing), then 30 s at 72 ^o^C (signal acquisition). Melting curve protocol was 1 min at 95 °C, followed by 30 s at 58 °C, then 0.11 °C/s to 95 °C followed by 10 s at 40 °C.

We used the LinRegPCR software package (version 2016.0; [5]) to correct for baseline fluorescence, set a window of linearity for each amplicon group and to calculate well-specific reaction efficiencies and Cq values. A constant fluorescence threshold was set within the window of linearity for each amplicon group, calculated using the average Cq across all three plates. The threshold values used were 0.140 and 0.203, and the average efficiency across all plates were 1.91 and 1.93 for the B2M and telomere amplicon groups, respectively. Samples were excluded from further analysis if the coefficient of variation (CV) across triplicate Cq values for either amplicon was > 5 %, or if at least one of their triplicate reactions had an efficiency that was 5 % higher or lower than the mean efficiency across all wells on that plate for the respective amplicon. Overall, thirteen samples failed quality control at either the DNA extraction or qPCR stage and were excluded from the study, leaving a total of 147 samples for further analyses.

We calculated relative telomere length (RTL) for each sample following Pfaffl et al 2001 as follows:

RTL = (E_TEL_^(CqTEL[Calibrator] – CqTEL[Sample])^)/ (E_B2M_ ^(CqB2M[Calibrator] – CqB2M[Sample])^)

Where E_TEL_ and E_B2M_ are the mean reaction efficiencies for the respective amplicon group across all samples on a given plate; CqTEL[Calibrator] and CqB2M[Calibrator] are the average Cqs for the relevant amplicon across all calibrator samples on the plate; and CqTEL[Sample] and CqB2M[Sample] are the average of the triplicate Cqs for the sample for each amplicon.

**Additional models testing the effect of body mass on telomere length**

To determine if and how variation in body condition within populations might influence our results, we generated metrics of body condition our best index of body size, by calculating the residuals from a linear model for body mass including an index of body size, hind foot length (n=133; b=0.231 ± 0.013 SE, p<0.001). We then tested for an association between size-corrected mass and TL, independent of previously identified associations between TL, age, sex and population, by adding size-corrected body mass to the minimal model for TL. There was no evidence for a relationship between TL and size-corrected body mass, and its inclusion in the TL model did not alter the magnitude of the age-by-population interaction (Fig. 1B; Table S3).

**SUPPLEMENTARY ETHICS**

The protocol of capture and blood sampling of roe deer under the authority of the Office National de la Chasse et de la Faune Sauvage (ONCFS) was approved by the Director of Food, Agriculture and Forest (Prefectoral order 2009-14 from Paris).

**SUPPLEMENTARY FUNDING**

Field and laboratory operations were supported by grants from the Agence Nationale de la Recherche (ANR-15-CE32-0002-01) and the ONCFS (grants 2011/18/6171 and 2015/19/6171), and performed within the framework of the LABEX ECOFECT (ANR-11-LABX-0048) of Université de Lyon, within the program “Investissements d’Avenir” (ANR-11-IDEX-0007).

**SUPPLEMENTARY REFERENCES**

[1] Gaillard, J.-M., Delorme, D., Jean-Marie, B., Van Laere, G., Boisaubert, B. & Pradel, R. 1993 Roe deer survival patterns: a comparative analysis of contrasting populations. *Journal of Animal Ecology*, 778-791.

[2] Hewison, A., Vincent, J., Angibault, J., Delorme, D., Laere, G.V. & Gaillard, J. 1999 Tests of estimation of age from tooth wear on roe deer of known age: variation within and among populations. *Canadian Journal of Zoology* **77**, 58-67.

[3] Seeker, L.A., Holland, R., Underwood, S., Fairlie, J., Psifidi, A., Ilska, J.J., Bagnall, A., Whitelaw, B., Coffey, M. & Banos, G. 2016 Method specific calibration corrects for DNA extraction method effects on relative telomere length measurements by quantitative PCR. *PLoS One* **11**, e0164046.

[4] Cawthon, R.M. 2002 Telomere measurement by quantitative PCR. *Nucleic Acids Research* **30**, e47. (doi:10.1093/nar/30.10.e47).

[5] Ruijter, J.M., Ramakers, C., Hoogaars, W.M.H., Karlen, Y., Bakker, O., van den Hoff, M.J.B. & Moorman, A.F.M. 2009 Amplification efficiency: linking baseline and bias in the analysis of quantitative PCR data. *Nucleic Acids Research* **37**, e45. (doi:10.1093/nar/gkp045).

**Table S1.** Linear models of telomere length in two populations of wild roe deer (N=139). (A) Maximal model including all terms and (B) minimal model after stepwise removal of non-significant effects. The reference groups for factors are females for sex and Chizé for population. Statistically significant terms in bold.

|  | *a ) RTL maximal model* | | | |  | *b ) RTL minimal model* | | | |
| --- | --- | --- | --- | --- | --- | --- | --- | --- | --- |
| **Explanatory variables** | **Estimate** | **SE** | **F-Value** | **P Value** |  | **Estimate** | **SE** | **F** | **P** |
| **Intercept** | **1.100** | **0.048** | **23.146** | **<0.001** |  | **1.072** | **0.035** | **30.548** | **<0.001** |
| Age | -0.012 | 0.007 | -1.623 | 0.107 |  | -0.011 | 0.006 | -1.660 | 0.106 |
| Population (TF) | -0.048 | 0.061 | -0.783 | 0.435 |  | -0.007 | 0.047 | -0.155 | 0.877 |
| Sex (males) | -0.037 | 0.058 | -0.643 | 0.521 |  |  |  |  |  |
| Age * Sex (males) | -0.004 | 0.010 | -0.392 | 0.696 |  |  |  |  |  |
| Sex (males) * Population (TF) | 0.060 | 0.060 | 0.996 | 0.321 |  |  |  |  |  |
| **Age * Population (TF)** | **0.026** | **0.009** | **2.718** | **0.007** |  | **0.022** | **0.009** | **2.509** | **0.013** |

**Table S2.** Linear models of body mass (kg) in two populations of wild roe deer (N=139). (A) Maximal model including all terms (B) minimal model after stepwise removal of non-significant terms. The reference groups for factors are females for sex and Chizé for population. Statistically significant terms in bold.

|  | *a ) Body mass maximal model* | | | |  | *b ) Body mass minimal model* | | | |
| --- | --- | --- | --- | --- | --- | --- | --- | --- | --- |
| **Explanatory variables** | **Estimate** | **Std Error** | **F-Value** | **P Value** |  | **Estimate** | **Std Error** | **F-Value** | **P Value** |
| **Intercept** | **11.133** | **1.015** | **10.966** | **<0.001** |  | **11.479** | **0.804** | **14.279** | **<0.001** |
| **Age** | **2.773** | **0.431** | **6.428** | **<0.001** |  | **2.543** | **0.342** | **7.436** | **<0.001** |
| **Age^2^** | **-0.175** | **0.037** | **-4.768** | **<0.001** |  | **-0.156** | **0.029** | **-5.339** | **<0.001** |
| **Population (TF)** | **3.818** | **1.188** | **3.215** | **0.002** |  | **3.161** | **0.454** | **6.963** | **<0.001** |
| Sex (males) | -0.795 | 1.212 | -0.656 | 0.513 |  | -0.539 | 1.043 | -0.517 | 0.606 |
| **Age * Sex (males)** | **1.293** | **0.520** | **2.487** | **0.014** |  | **1.238** | **0.511** | **2.424** | **0.017** |
| **Age^2^ * Sex (males)** | **-0.126** | **0.048** | **-2.622** | **0.010** |  | **-0.121** | **0.047** | **-2.568** | **0.011** |
| Sex (males) * Population (TF) | 0.301 | 0.942 | 0.319 | 0.750 |  |  |  |  |  |
| Age * Population (TF) | -0.463 | 0.509 | -0.911 | 0.364 |  |  |  |  |  |
| Age^2^ * Population (TF) | 0.040 | 0.045 | 0.881 | 0.380 |  |  |  |  |  |

**Table S3.** Linear models testing the effect of size-corrected body mass (n=133) on telomere length in two populations of wild roe deer. Residuals from models from a linear model of body mass containing only hind foot length as a covariate were added to the minimal model for RTL (see Table S1B). The reference groups for factors are females for sex and Chizé for population. Statistically significant terms in bold.

| **Explanatory variables** | **Estimate** | **Std Error** | **F-Value** | **P Value** |  |
| --- | --- | --- | --- | --- | --- |
| **Intercept** | **1.072** | **0.037** | **28.923** | **<0.001** |  |
| Age | -0.010 | 0.007 | -1.344 | 0.181 |  |
| Population (TF) | -0.018 | 0.048 | -0.379 | 0.705 |  |
| Size-corrected body mass | -0.002 | 0.007 | -0.331 | 0.741 |  |
| **Age * Population (TF)** | **0.024** | **0.009** | **2.572** | **0.011** |  |

**Figure S1.** Plot illustrating differences in body mass with age in the two study populations: the better environment at Trois Fountaines (TF, blue) and the poorer environment at Chize (CH, red). Raw data for CH (red) and TF (blue) are presented with a quadratic regression function (red and blue lines, respectively) along with their associated standard errors (grey shading).


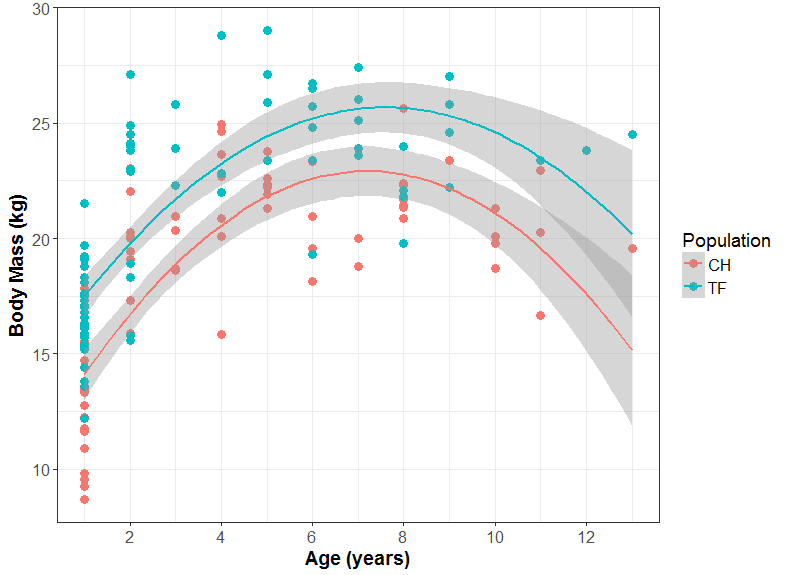

Supplement: Supplementary material [file rsbl20170434supp1.docx]
